# Simultaneous Detection of Forbidden Chemical Residues in Milk Using Dual-Label Time-Resolved Reverse Competitive Chemiluminescent Immunoassay Based on Amine Group Functionalized Surface

**DOI:** 10.1371/journal.pone.0109509

**Published:** 2014-10-14

**Authors:** Dongdong Zhang, Xiaoqi Tao, Haiyang Jiang, Kai Wen, Jianzhong Shen, Xingyuan Cao

**Affiliations:** 1 College of Veterinary Medicine, China Agricultural University, Beijing, China; 2 College of Food Science, Southwest University, Chongqing, China; CNR, Italy

## Abstract

In this study, a sensitive dual-label time-resolved reverse competitive chemiluminescent immunoassay was developed for simultaneous detection of chloramphenicol (CAP) and clenbuterol (CLE) in milk. The strategy was performed based on the distinction of the kinetic characteristics of horseradish peroxidase (HRP) and alkaline phosphatase (ALP) in chemiluminesecence (CL) systems and different orders of magnitude in HRP CL value for CAP and ALP CL value for CLE in the chemiluminescent immunoassay. Capture antibodies were covalently bound to the amine group functionalized chemiluminescent microtiter plate (MTP) for efficient binding of detection antibodies for the enzymes labeled CAP (HRP-CAP) and CLE (ALP-CLE). The CL signals were recorded at different time points by the automatic luminometers with significant distinction in the dynamic curves. When we considered the ALP CL value (about 10^5^) of CLE as background for HRP CL signal value (about 10^7^) of CAP, there was no interaction from ALP CL background of CLE and the differentiation of CAP and CLE can be easily achieved. The 50% inhibition concentration (IC_50_) values of CAP and CLE in milk samples were 0.00501 µg L^−1^ and 0.0128 µg L^−1^, with the ranges from 0.0003 µg L^−1^ to 0.0912 µg L^−1^ and from 0.00385 µg L^−1^ to 0.125 µg L^−1^, respectively. The developed method is more sensitive and of less duration than the commercial ELISA kits, suitable for simultaneous screening of CAP and CLE.

## Introduction

Feeding of drugs and chemicals to food animals (pigs, cattle, and goats) can leave residues in meat or milk, offal and other parts of the animals. With food poisoning cases and toxic effects arising from consumption of these residues and the emergence of drug resistant bacteria over recent years, there is a need to control the feeding of drugs and chemicals to the food animals [1]–[3]. A variety of analytical methods to detect and qualify drugs and chemicals in food matrices have been reported, such as gas chromatography–mass spectrometry (GC–MS), liquid chromatography (LC) with an iron trap detector and LC–MS, enzyme linked immunoassay (ELISA), surface plasmon resonance-based biosensor, immunoassay based on gold nanoparticles and magnetic beads, quantum dot-based lateral flow immunoassay, electrochemical biosensor, amperometric immunosensor and piezoelectric immunosensor [4]–[13]. Briefly, current trends for detection chemicals are divided to two directions: confirmatory methods such as GC-MS or LC-MS which could differentiate the chemicals and make multi-component detection, and rapid screening methods which satisfied the requirement of rapid and ‘Yes/NO’ at the level of interest for in-field controls. Monitoring the growing number of antibiotics, hormones, endocrine disrupting chemicals, toxins in animal-derived food samples places a high demand on rapid, cheap and reliable screening methods. Recently, increasing attention has been paid to the development of various multiplexed immunoassay (MIA) in a single run to achieve multi-residue determination and serve specific analytical purposes, which has been demonstrated to be promising in food safety, clinical diagnosis and environmental monitoring [14]–[17]. In this study, the goat anti-rabbit and goat anti-mouse immunoglobulins were bound covalently to the 3-aminopropyltriethoxysilane (APTES)-functionalized microtiter plates (MTP) in a leach-proof fashion using 1-ethyl-3-(3-dimethylaminopropyl) carbodiimide hydrochloride (EDC) and N-hydroxysulfosuccinimide (SNHS) to enhance the sensitivity of the MIA and reduce assay duration [18]–[20].

Chloramphenicol (CAP), a broad spectrum antibiotic, is frequently employed in animal production for its excellent antibacterial and pharmacokinetic properties. However, in humans it leads to hematotoxic side effects [2], in particular CAP-induced aplastic anaemia for which a dosage-effect relationship has not yet been established, leading to a prohibition of CAP for the treatment of food-producing animals [21]. Clenbuterol (CLE), a representative of the class of synthesized β_2_-adrenergic agonists, is abused illegally for food-producing animals as “lean meat agent”, which have potential hazard to human health. Hence, the development of an immunoassay format with enhanced analytical performance is essential for the precise simultaneous detection of trace CAP and CLE.

**Figure 1:**
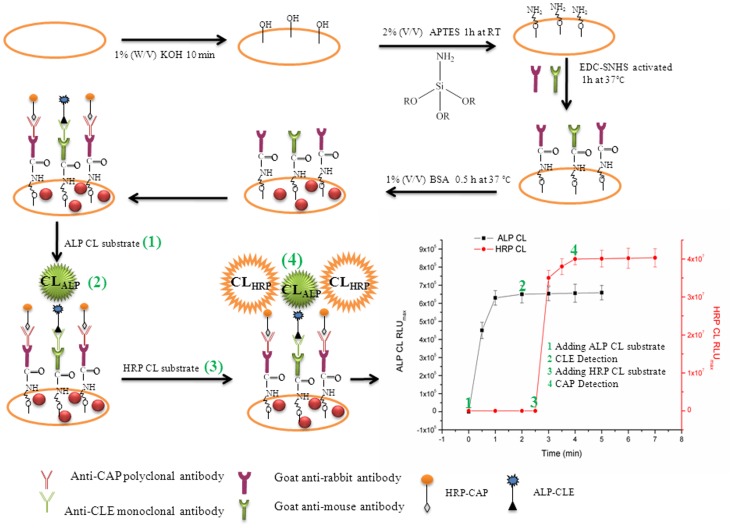
Schematic representation of DLTRRC-CIA for quantitative determination of CAP and CLE.

In this study, we have developed a sensitive rapid dual-label time-resolved reverse competitive chemiluminescent immunoassay (DLTRRC-CIA) for simultaneous determination of trace CAP and CLE applicable in milk. Figure 1 introduces the new assay design, which incorporates the advantages of different properties of chemiluminescence (CL) reaction (horseradish peroxidase (HRP) and alkaline phosphatase (ALP)) and sensitivity improvement of covalent binding. To date, the DLTRRC-CIA is the most sensitive assay with the least assay time for the simultaneous determination of trace CAP and CLE.

## Materials and Methods

### Materials and Reagents

(a) Standards—CAP (99% purity, Sigma-Aldrich, St. Louis, MO, USA); florfenicol (FF, 99%), florfenicol amine (FFA, 97.6%) and thiamphenicol (TAP, 97.6% purity) were puchased from Schering-Plough Corp. (Kenilworth, NJ, USA); CLE, salbutamol (SAL), ractopamine (RAC), sulfadiazine (SUL), ciprofloxacin (CIP), penicillin (PEN) were purchased from Shanghai Caienfu Technology Co. Ltd. (Shanghai, China).

(b) Analytical grade regents—EDC, SNHS, 2-(N-morpholino) ethanesulfonic acid (MES, pH 4.7), potassium hydroxide (KOH) and 3-APTES were purchased from Sigma (St. Louis, MO, USA). All other chemicals and organic solvents were of reagent grade and were from Beijing Chemical Co. (Beijing, China).

(c) The chemiluminescence Super Signal substrate solution was purchased from Pierce (Rockford, IL, USA). The Visiglo Plus AP Chemiluminescent Substrate was obtained from Invitrogen (NY, USA).

(d) The anti-CAP polyclonal antibodies (PAbs) (rabbit source), anti-CLE monoclonal antibody (MAb) (mouse source), HRP labeled CAP (CAP-HRP), ALP labeled CLE (CLE-ALP) were obtained from WDWK Biotech Co. (Beijing, China). Both of commercial conventional CAP ELISA kit and CLE ELISA kit were purchased from WDWK Biotech Co. (Beijing, China) and R-Biopharm (Beijing, China), respectively. Goat anti-rabbit immunoglobulins and goat anti-mouse immunoglobulins were from Sigma-Aldrich (St, Louis. MO, USA).

(e) Buffers and solutions were prepared in Milli-Q deionised water (DIW). Coating buffer (CB, pH 9.6) was made with 1.59 g Na_2_CO_3_ and 2.93 g NaHCO_3_ in 1 L of purified water. Blocking buffer was prepared by 0.01 M sodium phosphate-buffered saline (PBS) with 1% BSA, pH 7.4. 0.01 M Phosphate-buffered saline (PBS, pH 7.4) was prepared by dissolving 8.0 g NaCl, 0.2 g KCl, 0.24 g KH_2_PO_4_, and 3.63 g Na_2_HPO_4_·12H_2_O in 1 L of purified water. PBST contained 0.01 M PBS with 0.05% Tween-20. A 0.02 M sodium phosphate (PB, pH 7.2) was 1.1 g NaH_2_PO_4_·2H_2_O and 5.16 g Na_2_ HPO_4_·12H_2_O in 1 L of purified water. Solution Carrez A was 0.36 M K_4_Fe(CN)_6_·3H_2_O and solution Carrez B was 1.04 M ZnSO_4_·7H_2_O. The dilutions of KOH and 3-APTES were diluted in DIW. EDC and SNHS were reconstituted in 0.1 M MES buffer, pH 4.7.

### Apparatus

Chemiluminescence was measured with Veritas Microplate Luminometer (Turner BioSystems, Sunny Vale, CA, USA). The colorimetric-ELISA was made by Sunrise microtiter plate reader (TECAN, Groedig, Austria). Transparent 96-well microtiter ELISA plates for colorimetric assay and 96-well chemiluminescent white opaque MTP were purchased from Costar (Cambridge, MA, USA). All buffers were prepared using Milli-Q H_2_O system (18 MΩ/cm) (EMD Millipore Corporation, Belleria, MA, USA).

Covalent Binding of Capture Antibodies to the APTES-functionalized MTP

Each well of the 96-well MTP was treated with 100 µL of 1.0% (w/v) KOH at 37°C for 10 min followed by five DIW washes (300 µL/well). The KOH-treated wells were then functionalized with amino groups by incubating with 100 µL of 2% (v/v) APTES at room temperature (RT) for 1 h inside the fume cabinet. The amine-functionalized MTP was subsequently washed five times with DIW in order to remove excess unbound APTES from the surface. The 990 µL of goat anti-rabbit (2 µg mL^−1^) and goat anti-mouse immunoglobulins (2 µg mL^−1^) mixture (capture antibodies) was incubated with 10 µL of a pre-mixed solution of EDC (4 mg mL^−1^) and SNHS (11 mg mL^−1^) for 15 min at 37°C. The 100 µL of resulting EDC-SNHS cross-linked goat anti-rabbit and goat anti-mouse immunoglobulins solution was added to each of the APTES-functionalized wells and incubated for 1 h at 37°C. The goat anti-rabbit and goat anti-mouse immunoglobulins -bound wells were then washed five times with PBS (300 µL/well).

### The Procedure of DLTRRC-CIA for CAP and CLE

The goat anti-rabbit and goat anti-mouse immunoglobulins -bound MTP wells were blocked with 200 µL/well of blocking buffer at 37°C for 0.5 h, subsequently washed five times with PBST. The goat anti-rabbit and goat anti-mouse immunoglobulins -bound MTP wells were incubated with mixture of anti-CAP PAbs (1∶40000 dilution in PBST) and anti-CLE MAb (1∶40000 dilution in PBST) for 30 min at 37°C, and subsequently washed five times with PBST. Then 100 µL/well of standard (cocktail of CAP and CLE) in 0.02 M PB or sample solution was added, followed 50 µL/well mixture of CAP-HRP (1∶40000 dilution in PBST) and CLE-ALP (1∶10000 dilution in PBST). The competitive reaction was allowed to take place for 30 min at RT. After washing five times, the CL signal was measured using a chemiluminesence reader at 2 min after automatic addition of 100 µL/well Visiglo Plus AP Chemiluminescent Substrate in injector A and the results were expressed in relative light units (RLU). At 2.5 min, the HRP activity was revealed by automatically adding 100 µL/well of a freshly prepared substrate mixture of Super Signal substrate solution in injector B. The CL signal was measured using a chemiluminesence reader at 4 min and the results were expressed in RLU.

### Data Analysis

Standards and samples were run in quadruplicate wells, and mean chemiluminescence intensity values were divided by RLU_max_ (chemiluminescence intensity in the absence of analyte).The ratio is defined as B/B_0_. Standard curves were obtained by plotting B/B_0_ against the logarithm of analyte concentration and fitted to a four-parameter logistic equation using Origin (version 8.0, Microcal, Northampton, MA, USA) software packages
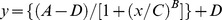
where A is the asymptotic maximum 1, B is the curve slope at the inflection point, C is the x value at the inflection point (corresponding to the analyte concentration that reduces RLU_max_ to 50%), and D is the asymptotic minimum (RLU_background_ _signal_/RLU_max_).

### Recovery and Precision

Standard solution were added into the blank milk samples, known to be free of CAP, and CLE, to yield CAP/CLE concentrations of 0.001/0, 0.0075/0, 0.04/0, 0/0.04, 0/0.020, 0/0.10, 0.001/0.004, 0.0075/0.020, 0.040/0.10 µg L^−1^, respectively. Measured concentrations with five times in duplicate for each sample to assess accuracy and precision.

### Analysis of Field Milk Samples

Forty whole cow milk samples with pakages were purchased from retail outlets in Beijing. Each sample was divided into three portions in brown polystyrene bottle, which would be analyzed by developed DLTRRC-CIA, conventional ELISA kits. All of the samples were stored at −20°C until use.

### Sample Preparation

For extraction of CAP and CLE from milk, added 500 µL Carrez A and 500 µL Carrez B to 10 mL milk, mixed thoroughly, then centrifuged for 10 min at 4000 g in 4°C. Transferred 4.4 mL of aqueous supernatant to a new tube, adjusted the pH to 11 with 1 M NaOH and thoroughly mixed with 8.0 mL ethyl acetate for 10 min in a new tube. Centrifuged at 4000 g for 10 min, 4 mL of organic supernatant was transferred to a new tube and dried by nitrogen at 60°C. The residue was dissolved in 2 mL of 0.02 M PB. The sample solution could be used for determination.

## Results and Discussion

### DLTRRC-CIA Strategy Based on Time-Resolved CL and Covalent Binding of Capture Antibodies

The DLTRRC-CIA for CAP and CLE based on time-resolved CL and covalent binding of capture antibodies is illustrated in Figure 1. HRP and ALP were adopted as the signal probes to tag CAP and CLE, respectively, due to their different CL kinetic characteristics. With a competitive immunoassay format, the HRP- and ALP-tagged immunocomplexes (goat anti-rabbit immunoglobulins—anti-CAP PAbs—CAP-HRP and goat anti-mouse immunoglobulins—anti-CLE MAb—CLE-ALP) were formed in the well of the MTP. The two CL signals were successively triggered by adding the two different CL substrates (ALP and HRP CL solutions were prepared in different injections in the automatic luminometer).

**Figure 2:**
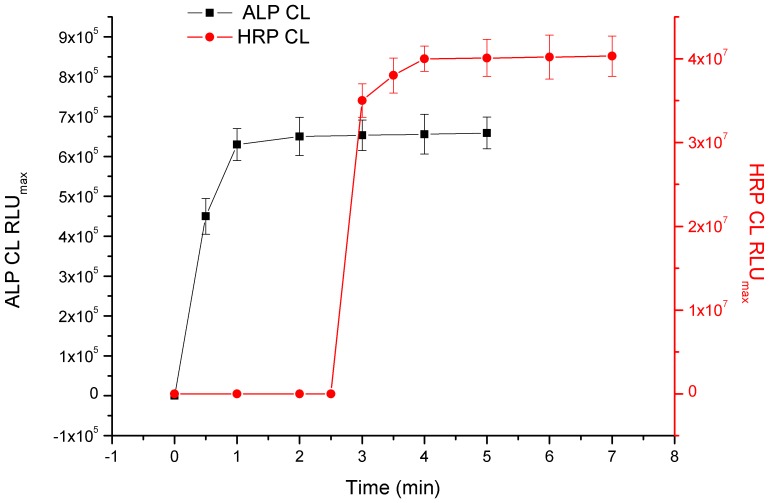
Kinetic measurement of chemiluminescence (CL) output intensity (RLU) for Super Signal and Visiglo Plus substrates catalyzed by HRP and ALP in the developed DLTRRC-CIA.

The CL signal of ALP-tagged immunocomplex reached the maximum value at 2 min after injection of ALP CL substrate in injector A, and showed a steady plateau in the whole measured period (Figure 2). The CL signal of the HRP-tagged immunocomplex reached the maximum value at 4 min after injection of HRP CL substrate in injector B (at 2.5 min), and showed a steady plateau in the whole measured period. Then the signal for CLE was detected at 2 min after adding ALP CL substrate (injector A) into the well. The signal for CAP was collected at 4 min after adding HRP CL substrate (injector B) into the wells at 2.5 min.

When the chemiluminesence reader collected the CL signal at 4 min, there were two chemiluminescent systems—HRP CL system for CAP and ALP CL system for CLE, and the CL value was the sum of HRP CL for CAP and ALP CL for CLE. But the ALP value of RLU_max-CLE_ was only 1/61 of RLU_max-CAP_ and the effect of ALP CL signal for CLE (0.65 million) on HRP CL signal for CAP (40 million) was negligible (Figure S1a). Thus we detected the signals of CAP at 2 min and CLE at 4 min, after the automatic injection of ALP CL substrate at 0 min and HRP CL substrate at 2.5 min. When CAP and CLE were both absent, the maximum HRP CL signal for CAP was 61 times of maximum ALP CL signal for CLE. Due to the distinguishable gap for HRP and ALP signals, the cross-talk resulting from the mixed CL reaction systems was effectively avoided (Materials S1 and Materials S2).

The APTES-functionalized bioanalytical platforms have been widely used for the leach-proof immobilization of biomolecules as they enable high immobilization density, long-term stability, high reproducibility, and less biofouling [19], [20], [22], [23]. In the present study, the covalent crosslinking of antibodies in a leach-proof manner on APTES-functionalized bioanalytical platform leads to high functional antibody immobilization density, which results in highly sensitive analyte detection and saving the assay duration (Table 1).

**Table 1:** A comparative analysis of the developed DLTRRC-CIA with various immunoassay formats and commercial kits for CAP and CLE detection in standard solution.

Manufacturer	Antibody binding	Sensitivity (µg L^−1^)	Assay duration (h)	Refer to
			CAP	
Developed DLTRRC-CIA	Covalently-bound	0.00501	5	Reported here
commercial CAP ELISA kits (WDWK Biotech)	Passively adsorbed	0.084	18	http://yu18710047045.foodmate.net/sell/index.php?itemid=27609
commercial CAP ELISA kits (R-Biopharm)	Passively adsorbed	0.082	18	http://www.xygen.com/pdfs/mycotoxins/antibiotics/Glucoronid-Cap.pdf
Direct competitive CL-ELISA	Passively adsorbed	0.017	18	[24]
Indirect competitive CL-ELISA	Passively adsorbed	0.13	>18	[25]

			CLE	
Developed DLTRRC-CIA	Covalently-bound	0.0128	5	Reported here
commercial CLE ELISA kits (WDWK Biotech)	Passively adsorbed	0.493	18	http://yu18710047045.foodmate.net/sell/index.php?itemid=21866
commercial CLE ELISA kits (R-Biopharm)	Passively adsorbed	0.472	18	http://www.bioon.com.cn/reagent/show_product.asp?id=934563
ELISA	Passively adsorbed	0.3	>18	[26]
Time-resolved CL immunoassay	Passively adsorbed	0.5	>18	[17]

### Performance of DLTRRC-CIA in Standard Solutions

**Figure 3:**
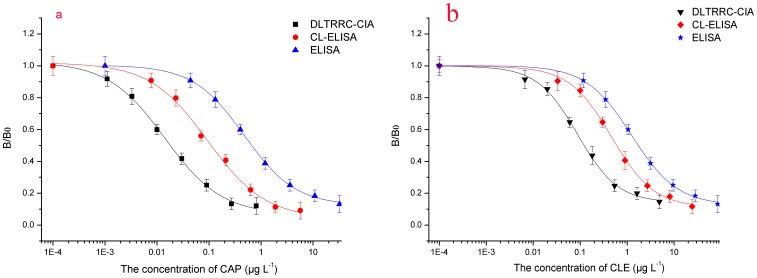
Normalized standard curve by developed DLTRRC-CIA for CAP (a) and CLE (b) under optimized conditions compared to the standard curve obtained by reverse competitive CL-ELISA and traditional ELISA method.

Under the optimal conditions, the CL responses decreased linearly with the increase in the concentrations of CAP and CLE since a competitive immunoassay mode was employed. The developed DLTRRC-CIA detected CAP and CLE in the range of 0.0003–0.0912 µg L^−1^ (R^2^ = 0.9971) and 0.00385–0.125 µg L^−1^ (R^2^ = 0.9974), with 50% inhibition concentration (IC_50_) values of 0.00501 and 0.0128 µg L^−1^, respectively (Figure 3). The sensitivity represented by IC_50_ of the developed DLTRRC-CIA for CAP were more than 9.2 times and 93 times greater than reverse competitive CL-ELISA (IC_50_ = 0.046 µg L^−1^) and traditional ELISA method with colorimetric detector (IC_50_ = 0.47 µg L^−1^) developed by our own, respectively (Figure 3a). Meanwhile, the sensitivity of the developed DLTRRC-CIA for CLE were more than 8 times and 96 times greater than reverse competitive CL-ELISA (IC_50_ = 0.11 µg L^−1^) and traditional ELISA method with colorimetric detector (IC_50_ = 1.25 µg L^−1^) developed by our own, respectively (Figure 3b). The detection limits represented by 10% inhibition concentration (IC_10_) values for CAP and CLE were 0.0003 and 0.001 µg L^−1^, respectively.

TAP, FF, FFA, SAL and RAC, structurally related with CAP or CLE, were selected for cross-reactivity (CR) experiments to evaluate the specificity of anti-CAP PAbs and anti-CLE MAb. No significant CR of anti-CAP PAbs to other amphenicols and β-adrenergic agonist was observed (Table 2). Meanwhile, no amphenicols and β-adrenergic agonists at concentration up to more than 1000 µg L^−1^ showed binding with the anti-CLE MAb. Furthermore, to help define the specificity of the two antibodies, structurally unrelated drugs including SUL, CIP and PEN were also tested. No CR was observed.

**Table 2:** CR of CAP and CLE in DLTRRC-CIA with some structurally related and unrelated compounds.

Compound	Structure	IC_50_ (µg L^−1^)	CR (%)
CAP	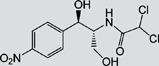	0.00501	100
TAP	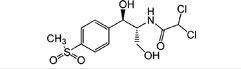	>1000	<0.1
FF	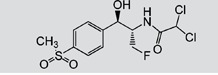	>1000	<0.1
FFA	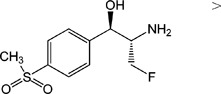	>1000	<0.1
CLE	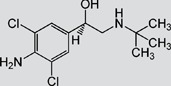	0.0128	100
SAL	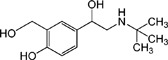	0.41	3.1
RAC	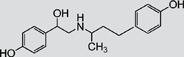	>1000	<0.1
SUL	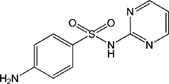	>1000	<0.1
CIP	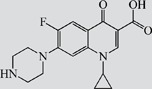	>1000	<0.1
PEN	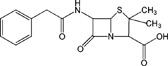	>1000	<0.1

### Comparison of the Developed DLTRRC-CIA for CAP and CLE with Other Immunoassays in Standard Solutions

The analytical comparison of various immunoassays for CAP and CLE detection is important to analyze their suitability for screening the residue in field samples. The developed DLTRRC-CIA decreased the overall assay duration significantly by more than 3-fold i.e. from 18 h (using the CL-ELISA procedure based on passively adsorbed anti-CAP PAbs, as employed in the commercial CAP kit) to about 5 h (Table 1). Its analytical sensitivity for CAP (0.00501 µg L^−1^) was 16-fold better than that of the commercial CAP ELISA kits (WDWK Biotech: 0.084 µg L^−1^; R-Biopharm: 0.082 µg L^−1^) and for CLE (0.0128 µg L^−1^) was about 36-fold better than that of the commercial CLE ELISA kits (WDWK Biotech: 0.493 µg L^−1^; R-Biopharm: 0.472 µg L^−1^). Moreover, it has better analytical performance, i.e. better analytical sensitivity with lower limit of detection (LOD), than our previously developed competitive direct CL-ELISA formats for CAP [24] and newly reported time-resolved chemiluminescence immunoassay for CLE [17], where the anti-CAP PAbs and anti-CLE MAb were passively adsorbed to the MTP, respectively.

### Matrix Effect

**Figure 4:**
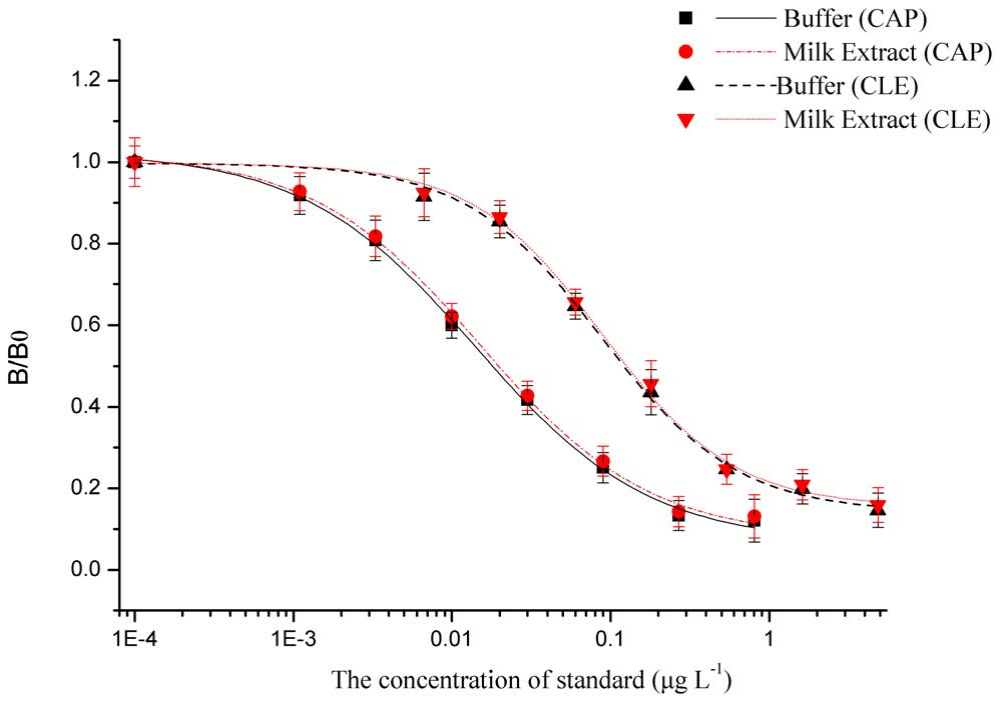
Inhibition curves of CAP and CLE in buffer and milk extract.

To apply a new method in real sample analysis, a matrix effect is an important issue to be considered, especially in animal tissues due to the complicated matrix. In this study, the established DLTRRC-CIA was used to determine CAP and CLE in field milk samples. When determining the matrix effects, interferences are quantified by comparing a standard inhibition curve in buffer with that generated in the milk extract matrix known to be free of CAP and CLE. Extracting with ethyl acetate, drying by nitrogen and dissolving in the same buffer with the standard curve buffer were adopted to overcome matrix interference. The two group curves for either of CAP and CLE are superposable, indicating that the matrix effect is not significant (Figure 4). Then the samples can be analyzed using the standard curve instead of the matrix curve.

### Application in Real Samples

#### Precision and recovery

Some spiked milk samples were detected using the developed DLTRRC-CIA method to evaluate the application potential. Five blank samples were spiked with CAP and CLE standards at different known amounts, prior to the recovery tests. As seen in Table 3, the recoveries of the spiked CAP and CLE were in the range of 85.5–91.5% and 83.7–89.6%, respectively. The coefficients of variation (CVs) for the detections of the two analytes were all less than 8.9%, indicating acceptable accuracy of the proposed method.

**Table 3:** Recovery of spiked CAP and CLE in milk.

	Spiked concentration	Measured	Measured		
Drug	(µg L^−1^)	(µg L^−1^)	(µg L^−1^)	Recovery	Recovery
	CAP/CLE	CAP	CLE	CAP	CLE
	0.001/0	0.0009±0.00005^a^	ND^b^	90.0%	ND
	0.0075/0	0.0067±0.0006	ND	89.3%	ND
	0.040/0	0.0366±0.0032	ND	91.5%	ND
CAP/CLE	0/0.004	ND	0.0035±0.0004	ND	87.5%
	0/0.020	ND	0.0179±0.002	ND	89.5%
	0/0.10	ND	0.085±0.026	ND	85.0%
	0.001/0.004	0.00085±0.0006	0.0036±0.0003	85.5%	89.4%
	0.0075/0.020	0.0068±0.0006	0.0177±0.0015	90.7%	88.6%
	0.040/0.10	0.0363±0.0034	0.089±0.0076	90.75%	89.0%

a Each value was repeated five times.

b Not detectable, <LOD.

#### Analysis of CAP and CLE in Field Milk Samples

To evaluate the determination capability of the developed DLTRRC-CIA in milk samples, 40 field samples were analyzed by the developed DLTRRC-CIA, conventional ELISA kits (Table 4). The results demonstrated that the developed DLTRRC-CIA could simultaneously screen CAP and CLE in the incurred samples as the ELISA kits did. Thereafter, the developed DLTRRC-CIA was reliable for the simultaneous screening of trace CAP and CLE residues in milk samples.

**Table 4:** Determination of milk samples collected from retail outlets in Beijing by the DLTRRC-CIA and traditional ELISA kit.

Sample	DLTRRC-CIA		Conventional ELISA kit (WDWK Biotech)		Conventional ELISA kit (R-Biopharm)	
	(µg L^−1^)		(µg L^−1^)		(µg L^−1^)	
	CAP	CLE	CAP	CLE	CAP	CLE
S2	0.074^a^	ND	0.072	ND b	0.076	ND
S4	ND	0.046	ND	ND	ND	0.045
S5	0.089	0.021	0.091	ND	0.090	ND
S11	ND	0.060	ND	ND	ND	0.058
S16	0.082	ND	0.079	ND	0.078	ND
S17	ND	0.060	ND	0.062	ND	0.067
S19	0.091	ND	0.11	ND	0.12	ND
S25	0.083	0.056	0.089	ND	0.090	0.054
S33	0.079	0.065	0.082	0.061	0.076	0.069
S1,S3, S6–S10,						
S12–S15, S18,			ND			
S20–S24,						
S26–S32,						
S34–S40						

a Each was determined with 3 repeats.

b ND not detectable.

## Conclusion

A novel sensitive DLTRRC-CIA was developed for simultaneous detection of CAP and CLE in milk based on distinguishable CL kinetic characteristics and covalent binding of capture antibodies. Since the adding substrate and detect time windows for HRP and ALP were different, and CL values for CAP (about 10^7^) and CLE (about 10^5^) were not in the same orders, the differentiation of CAP and CLE can be easily achieved. Goat anti-rabbit and goat anti-mouse immunoglobulins were covalently bound to the APTES-functionalized MTP, enhancing the sensitivity of the CIA for CAP and CLE, and reducing the assay duration. There was almost no cross-interaction resulting from the mixed CL reaction systems, testified by the recovery test. This strategy shows outstanding advantages such as low cost, high sensitivity, short assay duration and high efficiency. The recovery test and analysis in field samples demonstrates potential utility of the developed DLTRRC-CIA for clinical simultaneous screening of CAP and CLE in milk.

## Supporting Information

Figure S1 **Gross standard inhibition curves and revised standard inhibition curves in **Figure 1**.**
(TIF)

Materials S1 **Experimental materials.**
(DOC)

Materials S2 **Estimation of interaction effect.**
(DOC)
