# Supplementary material for: Simultaneous Detection of Forbidden Chemical Residues in Milk Using Dual-Label Time-Resolved Reverse Competitive Chemiluminescent Immunoassay Based on Amine Group Functionalized Surface
Source: PLoS One. 2014 Oct 14;9(10):e109509. doi: 10.1371/journal.pone.0109509 (PMC4196907; doi:10.1371/journal.pone.0109509)
Supplement: Materials S1 — Experimental materials. (DOC) [file pone.0109509.s002.doc]

**Materials S1**

**Experimental Materials**

*Standards*

The CAP, FF and FFA stock solutions were prepared in methanol; CLE, SAL, RAC and sulfadiazine were prepared in ethanol; ciprofloxacin and penicillin were prepared in purified water; TAP was prepared in dimethylfomamide. The stock solution (2 mg mL^−1^) was stored at -20 °C, and working standards were prepared from the stock solution by serial dilution in 0.02 M PB. Working standard (cocktail of CAP and CLE) in the range of 0.000167–0.3645 μg L^−1^ (CAP) and 0.004–0.8748 μg L^−1^ (CLE) were prepared from the 2 mg mL^-1^ stock solution by serial dilution in 0.02 M PB.

*Buffers*

The following buffers were used:

(a) Coating buffer (CB, pH 9.6)—0.05 M carbonate/bicarbonate buffer was made with 1.59 g Na_2_CO_3_ and 2.93 g NaHCO_3_ in 1 L of purified water.

(b) Blocking buffer was prepared by 0.01 M sodium phosphate-buffered saline (PBS) with 1% BSA, pH 7.4

(c) Phosphate-buffered saline (PBS, pH 7.4)—0.01M PBS was prepared by dissolving 8.0 g NaCl, 0.2 g KCl, 0.24 g KH_2_PO_4_, and 3.63 g Na_2_HPO_4_·12H_2_O in 1 L of purified water.

(d) PBST contained 0.01 M PBS with 0.05% Tween-20.

(e) A 0.02 M sodium phosphate (PB, pH 7.2) was 1.1 g NaH_2_PO_4_·2H_2_O and 5.16 g Na_2_ HPO_4_·12H_2_O in 1 L of purified water.

(f) Carrez A: 0.36 M K_4_Fe(CN)_6_·3H_2_O; Carrez B 1.04 M ZnSO_4_·7H_2_O.
