# Supplementary material for: Simultaneous Detection of Forbidden Chemical Residues in Milk Using Dual-Label Time-Resolved Reverse Competitive Chemiluminescent Immunoassay Based on Amine Group Functionalized Surface
Source: PLoS One. 2014 Oct 14;9(10):e109509. doi: 10.1371/journal.pone.0109509 (PMC4196907; doi:10.1371/journal.pone.0109509)
Supplement: Materials S2 — Estimation of interaction effect. (DOC) [file pone.0109509.s003.doc]

**Materials S2**

Estimation of interaction effect

In this strategy, the HRP CL was based on the reaction of luminol and H_2_O_2_ catalyzed by HRP, and ALP CL was based on the ALP chemiluminescent substrate (disodium 3-(4-methoxy-spiro{1, 2-dioxetane-3, 2′-(5′-chloro) tricycle [3.3.1.1 ^3,7^] decan}-4-yl) phenyl phosphate (CSPD)). Thus, the reaction of two systems will not interfere with each other. Moreover, the HRP and ALP CL signals were resolved in different time windows. The ALP substrate for the ALP-tagged immunocomplex was automatically added at 0 min and the ALP CL signal was measured at 2 min. The HRP substrate for HRP-tagged immunocomplex was automatically injected at 2.5 min and the HRP CL signal was measured at 4 min.

Although there were two CL signals from HRP-tagged immunocomplexes (goat anti-rabbit immunoglobulins—anti-CAP PAbs—CAP-HRP) and ALP-tagged immunocomplex (goat anti-mouse immunoglobulins—anti-CLE MAb—CLE-ALP) when the CL value was measured at 4 min, the maximum CL value for CAP was 61 times of that for CLE. Furthermore, the minimum CL value at maximum CAP concentration (0.8748 μg L^−1^) was about 7.4 time of maximum CL value for CLE (Concentration: 0 μg L^−1^) (Supplementary Figure 1a). When we considered the ALP CL value for CLE as background for the HRP CL signal value for CAP and subtracted the background (maximum ALP CL value for CLE) from the HRP CL value for CAP at different concentrations, a revised standard curve was obtained (Supplementary Figure 1b). The almost identical inhibition curves suggested that there was almost no interaction effect from ALP CL background of CLE. Meanwhile, when CAP or CLE was individually spiked in milk at different concentrations, respectively, the recovery ranged from 83.7%-91.5%. When CAP and CLE were co-spiked in milk at different concentrations, the recovery ranged from 85.5%-90.9%. In a word, the good recovery and insignificant difference of recovery between the individual spiked and co-spiked groups at the same fortified concentration indicated that the interaction effect from the mixed CL reaction was effectively avoided.
